# Avicenna’s Concepts on Cancer Metastasis from the 11^th^ Century

**DOI:** 10.31661/gmj.v8i0.1292

**Published:** 2019-01-01

**Authors:** Sina Kardeh, Bahareh Kardeh

**Affiliations:** ^1^Cellular and Molecular Medicine Student Research Group, Medical School, Shiraz University of Medical Sciences, Shiraz, Iran; ^2^Student Research Committee, Shiraz University of Medical Sciences, Shiraz, Iran; ^3^Bone and Joint Diseases Research Center, Clinical Neurology Research Center, Shiraz University of Medical Sciences, Shiraz, Iran

**Keywords:** Avicenna, Breast, Cancer, Metastasis

## Abstract

From ancient times to the era of industrialization, medical and philosophical scholars have long been wondering about the nature and the underlying mystery of cancer. Despite the extensive efforts in recent decades trying to shed light on the true histopathologic basis of malignancies, many questions remain to be elucidated. Thus, it’s not surprising that even the most notable predecessor physicians knew only very little about cancer and metastasis. In this paper, we present a brief review of the historical assumptions about the origin and spread of tumoral growths. Subsequently, we will look at an impressing notion by Avicenna about the possibility of local metastasis, which had remained unacknowledged so far and highlights the intellect of this great Persian physician even further.

## Introduction

Cancer refers to a large group of disease entities characterized by abnormal development of numerous cells that divide disorderly and have the ability to infiltrate other tissues and cause destruction [1]. Both developed and developing countries are burdened by cancer with a rising rate as a major cause of morbidity and mortality. This phenomenon is largely due to population aging as well as risk factors related to an unhealthy lifestyle such as smoking, overweight, and urbanization [2]. Unfortunately, one-third to half of all cancers are correlated with preventable risk factors [3]. This is of note that although the sheer cancer prevalence in the developed world is two times greater in comparison to the developing countries, the mortality rate is only 8-15% higher. This discrepancy can be explained by the differences in cancer types, infection-related cancers, stage of detection, and the feasibility of proper treatments [4]. In spite of its highly elevated frequency in the modern era, cancer has attracted attention since a long time ago as there are pieces of evidence even in ancient inscriptions that refer to benignand malignant tumors. In this historical review, we provide an insight about the evolution of knowledge surrounding cancer, and for the first time highlight the fact that Avicenna was the first scholar to introduce the concept of local metastasis.

### Cancer throughout History; Notable Milestones

The oldest statement about cancer dates back to 3000 BC in the Edwin Smith papyrus, which contains a description of breast cancer and suggests the disease has no treatment. Enlarged thyroids, polyps, skin, uterus, pharynx, stomach, and rectal tumors have also been mentioned in the Ebers papyrus from 1500 BC [5]. Even the Ramayana, an Indian epic, mentions malignant diseases and their treatments [5]. Although there are several ancient reports on cancer or malignant tumors, most are based on gross diagnoses. However, microscopic reports are also available, such as the detection of small squamous papilloma by Sandison and the diagnosis of dermatofibroma by Zimmerman in mummies [6]. Therefore, it is expected that cancer must have been rare in previous times because mankind did not experience the longevity and the considerable exposure to carcinogens like he does today. Yet, the antiquity of cancer cannot be denied. Hippocrates (460–375 BC) had several references to cancer in his documentation. He realized that growing tumors mainly afflict the adults [7]. Also, the nomenclature of oncology is built upon his comparison of tumoral growths with a moving crab, which led to the generation of scientific terms such as cancer (a non-healing malignant ulcer) and carcinoma (a malignant tumor) [8]. He theorized that an imbalance in body humor, i.e. blood, phlegm, yellow bile, and black bile instigates diseases [7] and that excess black bile is responsible for the development of cancer [9]. The humoral theory, which considered cancer a general disease, was the standard of medical practice through centuries because autopsies were banned due to religious prohibitions and also because its theoretical nature would leave no questions unanswered [10]. Galen (131 – 200 AD), one of the undoubtedly most influential physicians for many centuries, adopted the humoral theory but believed that the black bile was responsible for incurable cancer, whereas thin bile was related to curable cancer [11]. Throughout the Islamic Golden Age (7th to 14th century), prominent Muslim Persian and Arab scholars penned about cancer in their writings [9]. Al Zahrawi (936–1013 AD) was the first to distinguish between acute kidney inflammation and kidney cancer [9]. Cancer was assumed to have a difficult treatment, which could be probably successful only if performed at an early stage and also if the tumor was accessible, small-sized, and not adjacent to major organs for the possibility of surgical removal. Ibn Sina (980-1037 AD), known as Avicenna in the west, was one of the leading pioneers of medical science in the Islamic Golden Age [12]. He clarifies his surgical approach to early removal of a tumoral growth in his eminent work “*Al-Qanun-fi-al-Tibb*” (The Canon of Medicine): “All diseased tissue should be removed with radical excision, which could utilize amputation and removal of veins surrounding the growth, or catheterization if necessary” [9]

.

### The Emergence of Metastasis Concept

More than 90% of cancer mortality is due to metastasis; i.e., the extension of malignancy from its primary site to other organs. The occurrence of metastasis depends on the aggressiveness of cancerous cells and their dissemination routes [13]. Also, target organs vary depending on the originating organs; for instance, cutaneous metastasis of breast cancer is more likely to invade the chest wall [14]. In antiquity, death could have been the direct or indirect result of the primary lesions; thereby, making detectable signs of metastasis even more scarce [15]. Nicolas Abraham studied the natural course of cancer and made an observation of delitescence spreading to the internal organs in the late 16th century [16]. Tissot applied the word “metastasis” as the transference of a virulent agent from one part of the body to another, though it mainly referred to infections rather than cancer [16]. LeDran (1685–1770) was the pioneer to recognize the local nature of cancer. He noticed that cancer agents were not limited to the primary site, rather they were also present in “cancer juices” that had the ability to spread. Accordingly, he associated the enlargement of lymph nodes with poor prognosis and therefore accompanied lymph node removal with the procedure of mastectomy [16]. At the end of 18th century, Matthew Baillie clearly discussed co-existing nodules in the liver of a patient with colon cancer. Yet, he failed to declare any links between the primary colon cancer and the metastasized liver [16]. Récamier used the term “metastasis” to refer to the spread of cancer for the first time in his book, which was published in 1829. In addition, he believed that metastasis was not the only way that cancer could spread. He introduced the interconnection of organs by nerves as another possible explanation [16]. By the early 19th century the nature of metastasis had been identified properly; however, a consistent explanation had not been established up to that time. The fundamental relationship between metastasis and a primary cancer site was asserted by Walshe in 1840s: “Cancer originates in one tissue and tends to spread, and affects other tissues by secondary cancer. How the tumor disseminates is dependent on the distance of the secondary organs. This may occur through lymphatic or venous systems.” [16].

### Ibn Sina’s Novel Notion of Local Metastasis

Avicenna cites a case of breast cancer and reflects his idea about the progression of the disease in the Canon of Medicine [9]. This outstanding medical encyclopedia was translated into Latin by Gerard of Cremona in the 12th century and remained a major medical reference textbook until the 16th century in the Western countries [9, 17]. Herein, we review Avicenna’s presentation of his case and his hypothesis: “One of the practitioners in the past once narrated that after a physician conducted radical excision on a cancerous breast, cancer advanced to the other breast. My opinion about this case is that the second breast might have been in the process of cancerization and when the physician excised the first breast, coincidentally, the second one developed cancer symptoms. However, a reasonable theory for this case that I think is more appropriate is that cancer material advanced to the second breast from the first breast (before it was excised) or other sources (afflicted tissues).” [18].

## Discussion

Although there have been several remarks about the spread of cancer, there is no documented statement about the spread of cancer from a local site to other regions of the body prior to the 15th century. Most physicians believed cancer was a general disease that could afflict any part of the body independent of the primary site. In contrast, Avicenna acknowledges the cancerous disease of the first breast and concludes that despite its excision, the cancerous material had already been seeded into the second (initially healthy) breast. Contrary to the previous and many succeeding physicians, he proposed that secondary cancer can merely originate due to the cancer material transferred from the primary site. The other fascinating point is that Avicenna developed his idea through studying the clinical course of a patient with breast malignancy, rather than seeking a theoretical reasoning. This approach is closer to scientific methods and can be perceived as his immense genius.

## Conclusion

It seems that Avicenna was the first scholar to describe the possibility of local metastasis from a primary cancerous source to surrounding tissues. Interestingly, his hypothesis was based on the clinical course of the patient rather than a theoretical justification.

## Acknowledgment

The authors would like to thank the Research Consultation Center of Shiraz University of Medical Sciences, Shiraz, Iran for proofreading this manuscript.

## Conflict of Interest

The authors declare no conflict of interest.
